# Oral health related quality of life in head and neck cancer survivors within the first year following treatment: a cross-sectional study in Karachi, Pakistan

**DOI:** 10.1038/s41598-024-52813-x

**Published:** 2024-01-31

**Authors:** Sana Qamar, Shafquat Rozi, Sobia Sawani, Muhammad Sohail Awan, Shabbir Akhtar, Moghira Iqbaluddin Siddiqui, Syed Akbar Abbas, Shazia Taimoor, Farhan Raza Khan

**Affiliations:** 1 Department of Community Health Sciences, Aga Khan University Hospital, Karachi, Pakistan; 2 Department of Surgery, Section of Otolaryngology and Head and neck Surgery, Aga Khan University Hospital, Karachi, Pakistan; 3 Department of Surgery, Associate of Science of Dental Hygiene, Aga Khan University Hospital, Karachi, Pakistan; 4 Department of Surgery, Section of Dental Surgery, Aga Khan University Hospital, Karachi, Pakistan

**Keywords:** Cancer, Health care, Medical research

## Abstract

After completing treatment for head and neck cancer (HNC), patients often face oral complications like oral pain, limited mouth opening and dry mouth which significantly reduce their oral health related quality of life (OHRQoL). These issues impact their overall well-being, social activities and long-term survival. The primary objective of this study was to evaluate OHRQoL and its association with sociodemographic characteristics, oral hygiene practices and oral clinical parameters such as oral hygiene status and oral mucositis grade in patients who have completed treatment for head and neck cancer. This cross-sectional study involved 79 HNC-treated patients within first year after completion of cancer treatment attending ENT and dental clinics at outpatient department (OPD) setting in Karachi. Data was collected electronically using structured questionnaire comprising of EORTC QLQ H&N – 35 to measure OHRQoL, patients were also examined for oral hygiene status using oral hygiene index- simplified (OHI-s) and oral mucositis grade using WHO oral mucositis scale. Multiple linear regression was used to test OHRQoL associations with the sociodemographic and different clinical factors. The result showed an overall mean score for oral health related quality of life (OHRQoL) of 25.02 ± 15.86 (95% CI 21.46–28.57), with difficulty in mouth opening 53.16 ± 18.88 and dry mouth 45.14 ± 24.48 being predominant concerns for decline in the OHRQoL in the population. Male predilection was observed among participants n = 60 (75.9%), majority of the participants n = 41 (51.9%) were below 52 years of age. n = 63 (80%) participants received radiotherapy alongside surgery and chemotherapy. Most of participants n = 66 (83.5%) experienced moderate to severe oral mucositis with poor oral hygiene status n = 56 (71%). Significant associations were found between OHRQoL and BMI, OH status, marital status, monthly income, gender and fluoride toothpaste (p < 0.05). These findings suggest that Quality of Life (QoL) among HNC treated patients is negatively impacted by their poor oral health, post cancer treatment. Therefore, it is important to evaluate and modify the current treatment modalities and involve multidisciplinary teams, to improve their OHRQoL thereby enhancing overall QoL.

## Introduction

Head and neck cancer (HNC) is the second most frequently occurring cancer in Pakistan, yet it is vastly underestimated, with only 16,595 reported cases annually^1,2^. Pakistan is among the top ten countries known for tobacco consumption, with 46% of its population consuming paan and gutka (chewable betel) in their daily lives^3^. Regions commonly involved in HNC are the oral cavity, tongue, oropharynx, nasopharynx, tonsils, and parotid gland^4^. Treatment choices for HNC such as surgical resection, radiotherapy (RT) and chemotherapy (CT) vastly depend on the stage of the tumor at the time of diagnosis. For advanced stages of cancer, the use of high-energy radiation can cause certain impairments in the oral cavity that can be severe, such as xerostomia due to reduced salivary secretions, mouth soreness, bacterial and fungal infections, taste disturbance, dysphagia, pain, bleeding from periodontal tissues, and speech problems^5,6^. Almost 56–80% of the HNC patients often report of having disturbed oral functions leading to various social, economic, and psychological consequences that profoundly impact their overall quality of life (QoL)^7^.

Due to the aggressiveness of the disease (cancer) as well as its available treatment, patients often suffer harsh side effects (pain, inflammation and systemic fatigue) impairing their willingness to maintain oral hygiene. Absence of regular brushing and interdental cleaning can result in microbial buildup^8^, also the reduction in the salivary flow following the RT/CT required for the self-cleansing of the oral cavity further aggravates the oral symptoms predisposing patients to oral pain, soreness, periodontal diseases and dental cavities^9^. Poor oral hygiene is not only the cause of oral diseases but studies have reported, strong association with systemic disorders such as hypertension, diabetes, kidney disease^10–12^. A study concluded that lack of oral hygiene especially in the cancer patients can be the result of their reduced motivational drive and lack of oral care education by the consulting physician^8^.

Oral mucositis (OM) is a common acute complication that typically arises around fourth week of radiotherapy (RT). Nearly 80–100% HNC patients report varying degrees of oral mucositis within first three months of treatment^13,14^. Lesions of oral mucositis are intensely painful, hindering chewing, swallowing and oral hygiene practices. Severe mucositis can result in malnutrition^15^, necessitating invasive interventions like nasogastric intubation or percutaneous endoscopic gastrostomy^16^. These interventions not only increase healthcare costs but also reduce QoL due to the associated risks of infection and extended hospital stay. It is worth noting that these symptoms are temporary, lasting for weeks or months after therapy completion^17^. Several studies have reported patients having chronic oral mucositis 6 months after RT and a longer prevalence of OM in patients receiving concurrent chemo-radiotherapy^18,19^. QoL assessment is an important tool for evaluating not only the impact of disease and its treatment on an individual level but also helpful in developing and revising the rehabilitative services and patient education material to further improve the clinical outcomes (survival) of patients^20^. Oral health related quality of life (OHRQoL), specifically focuses on self-report aspects pertaining to oral health, capturing the functional, social, and psychological dimensions affected by oral disease^21^. In the case of HNC, disruptions in key oral functions poses a higher risk of adversely affecting both OHRQoL and overall QoL.

**Figure 1:**
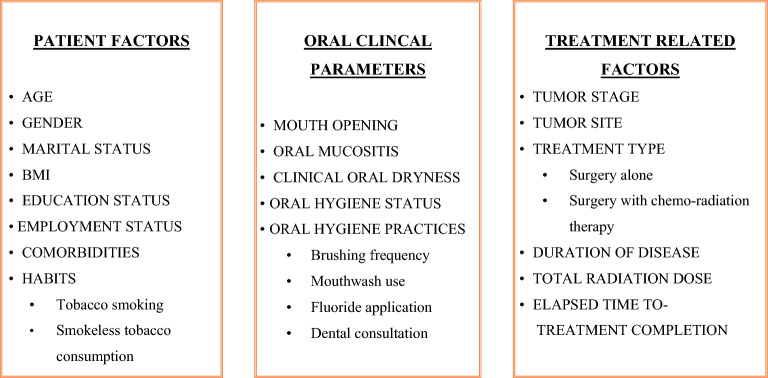
Factors affecting the OHRQoL among HNC patients, post cancer treatment.

Not much evidence is found in Pakistan related to oral care measures for HNC patients, such as professional care by a dentist and dental hygienist (mechanical or manual removal of mucosal debris, oral hygiene counselling, etc.) at least once a week until the completion of RT^19^. The patients are often unaware of the potential harm from the cancer treatment, only a proportion of HNC patients seek dental consultation for their complications, and most of them suffer greatly even after the completion of treatment due to disturbed oral functions. To the best of our knowledge, this study represents the first comprehensive assessment of OHRQoL in HNC treated patients who had completed their cancer treatment within the past 12 months. Additionally, it aims to explore the associations between various oral clinical parameters, including oral hygiene status and oral mucositis, and several predictive factors such as sociodemographic characteristics and treatment-related variables in relation to OHRQoL (see Fig. 1). The findings of this study highlights the importance of standardizing oral care and improving oral health support for HNC treated patients to reduce the impact of oral problems which can negatively impact their overall quality of life.

## Material and methods

### Study design and study setting

A cross sectional study was conducted in outpatient department of ENT and Dental clinics at Aga Khan University Hospital (AKUH), recognized as the largest private tertiary care hospital in Karachi, Pakistan accredited by Joint Commission International (JCi) This facility provides comprehensive care to cancer patients serving diverse ethnic and socioeconomic backgrounds, and has benefitted more than 3 million cancer patients to date^22^. Additionally, AKUH actively promotes research activities and maintains a well-organized patient registry.

### Study population, sampling technique and sample size

The participants in this study were patients who were 18 years and above, treated for HNC at Aga Khan University Hospital (AKUH). The study specifically included HNC survivors who were within the first year after completing their cancer treatment^23^, coming for their routine follow up at ENT and Dental clinics at AKUH. All the included patients had one of the three molars and a central incisor in upper and lower dentition at the time of follow up (these teeth were required to measure the Oral hygiene status using Oral Hygiene Index- simplified). We excluded patients who didn’t give their consent to participate in the study or had any debilitative conditions or cognitive disabilities confirmed by their medical reports or had trismus i.e. mouth opening < 20 mm (as it hindered the oral examination of the participant)^22,24^.

Participants were recruited using a non-probability purposive sampling technique. The study aimed to achieve its objectives with a minimum sample of 76 HNC-treated patients, considering an 80% statistical power and a significance level of 0.05. adjustments were made considering a 10% non-response rate, with an anticipated mean score 5 units higher than the hypothesized value of 16.7 in our population, as advised by the subject specialist^19^.

**Figure 2:**
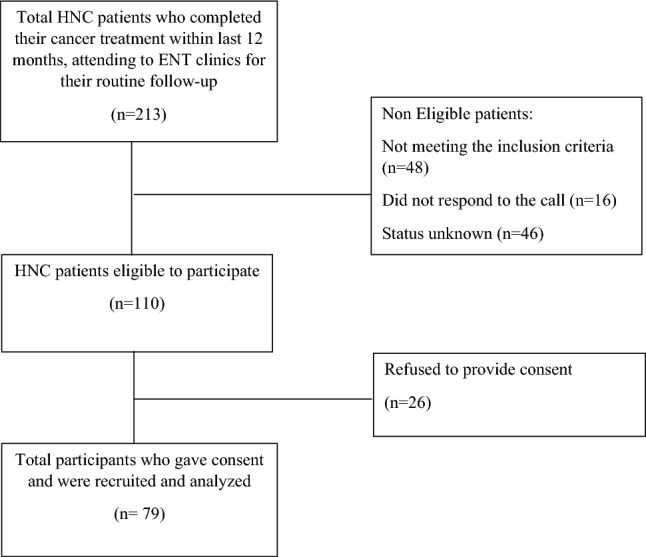
Flowchart of the process of study participant’s recruitment.

All patients treated for HNC at AKUH, between January 2021 and September 2022 were screened via their medical records. Patients who were within their first year of post-cancer treatment were contacted by phone to inquire about their upcoming follow-up visit to the ENT or Dental clinics at AKUH. On the scheduled follow-up day, we approached these patients to check for eligibility (i.e. for mouth opening and presence of at least one molar and incisor in each quadrant). Eligible patients were invited to participate in the study after explaining the purpose and scope of the study. Those who provided their written consent were interviewed and examined (Fig. 2). This study was approved by the Ethical review committee of Aga Khan University (2022-7178-21416).

### Variables and tools

Data was collected through a structured questionnaire, comprising sociodemographic variables, treatment related factors, oral hygiene practices and oral clinical parameters (oral mucositis and oral hygiene status).

### Demographic and other variables

Sociodemographic variables included patients age recorded as (< 52 years’ vs ≥ 52 years)^25^, gender, marital status, BMI recorded as (underweight ≤ 18.5 kg/ m^2^, normal weight =  ≥ 18.5 kg/ m^2^)^26^, level of education and monthly income. Treatment related factors included tumor stage at diagnosis according to AJCC stage system (stage I, stage II, stage III, stage IV), type of treatment received (without RT vs with RT), total dose of radiation received, elapsed time since treatment (< 6 months’ vs ≥ 6 months), number of follow up visits and any comorbidities Patients oral hygiene-related details included their practices of brushing and mouthwash use and frequency per day, use of fluoride toothpaste, any instructions received related to oral hygiene maintenance after treatment completion by the consultant, and the current use of dental prostheses.

Oral clinical parameters such as Oral Hygiene status was measured using Oral Hygiene Index – simplified (OHI-S) and categorized as good (0–1.2), fair (1.3- 3.0) and poor (3.1–6.0)^27^. The Oral Mucositis was assessed using WHO- Oral mucositis scale, and categorized into mild (by combining grade 1 and 2) and moderate to severe (by combining grade 3 and 4) categories^28^.

### Outcome variable

OHRQoL is characterized as a self-report measure that focuses specifically on the oral health. it encompasses a comprehensive assessment of how oral diseases and conditions impact various aspects of an individual’s life. It assess the functionality as well as social and psychological effects stemming from oral health issues, aiming to understand their overall impact on well-being^21^. For this study we used Urdu version of European Organization of Treatment of Cancer QoL Head and Neck-35 (EORTC QLQ-H&N-35), which has been validated for use in our setting^24^.

EORTC QLQ-H&N-35 consists 35 questions in total, with 7 multiple item- questions assessing pain and soreness in the mouth and throat (PA) through item 31 -34, swallowing of liquid, pureed and solid food (SW) through item 35–38, senses of taste and smell (SE) from 43–44, social eating with family and friends and enjoying of meal (SO) 49–52 social contact with family and friends (SC) 48, 55–58 and sexuality (SX) 59–60 over the last week and 11 single questions to assess problems with “teeth” “opening of mouth” “dry mouth” ” sticky saliva” “coughing” “feeling ill” “ use of pain killers” “nutritional supplements” or “feeding tube” “weight loss” and “weight gain”. Likert type was used to score the responses as (1 = not at all, 2 = a little, 3 = quite a bit, 4 = very much) item H&N31 – H&N-35 have a 1 = yes, 2 = no response^29^.

### Scoring of tool

First raw scores were calculated. For each multi- item question such as PA, SW, SO etc. an average of the corresponding items and for single- item question (teeth, mouth opening, sticky saliva, dry mouth etc.) the single score of the concerning item was taken as the raw score. *Raw score* = *(I*_*1*_ + *I*_*2*_ + *I*_*3*_*)/n.*

Standardization of the raw score was done to obtain mean scores for OHRQoL ranging from 0 to 100. *Symptom scale S* = *[(RS-1)/range]* × *100.*

Elevated symptom scores for different domains of the tool implied deteriorating oral symptoms contributing to overall high mean score indicating more adversely affected OHRQoL.

### Data collection procedure

Hiring of data collector was done prior to data collection. The training of data collector included procedure of taking informed consent, familiarization with the questionnaire and time management. A manual of operations was provided to data collector to facilitate consistency in protocol implementation during data collection. A trained dental hygienist was recruited to perform oral examination of the participants after they were interviewed.

### Ethical clearance

An ethical review was sought from the Departmental Review Board (DRC), after its approval, the Ethical Review Committee (ERC) of Aga Khan University, Karachi, was consulted for ethical approval. The ERC approved the study (reference number 2022-7178-21416).

### Statistical analysis

OHRQoL, which is the outcome variable is continuous in nature and calculated on the basis of a mean score. Multiple linear regression analysis was performed using STATA version 16.

The descriptive statistics of all independent variables and outcome variable was conducted. For categorical variables such as age, gender, level of education, monthly income, oral hygiene practices and other variables, frequencies and percentages were reported.

Normality of the outcome i.e. OHRQoL was checked using normal probability plot, which showed normal distribution. At the univariate analysis stage, all the categorical variables i.e. age, gender, marital status, BMI, educational status, employment status, monthly income, tumor stage, treatment type, radiation dose, number of follow up, smoking status and frequency, brushing and mouth wash use and their frequency, use of fluoride tooth paste, guidance related to oral complications of cancer treatment, visit to dentist, use of dental prosthesis were individually assessed for a significant association with OHRQoL on a set P value of ≤ 0.25.Variables with p-value greater, were removed and other variables with significant p-values were included in multivariable analysis. In Multivariable analysis, all variables that were significant at univariate analysis level, were assessed using multiple linear regression analysis. All variables with a p-value ≤ 0.05 were added in the model. The presence of biologically plausible interaction and confounding was also assessed.

After finalization of the main effect model, adequacy was assessed using residual plots against fitted values and normal probability plot.

### Data management

Data was collected by a web-based structured questionnaire on google forms, which included mandatory questions, thereby, eliminating the issue of missing or incomplete data. The data was assessed for accuracy and completeness on daily basis by the investigator. Built-in range checks and internal consistency checks in google forms further ensured data quality. Backup files were created for data security, with participant information (such as name and contact details) excluded to maintain confidentiality. Each participant was assigned a unique ID number for data organization. The electronic data has been password- protected and will be deleted after seven years as per the policy of Aga Khan University.

### Ethics approval and consent to participate

Ethical approval was obtained from Ethical Review Committee of the Aga Khan University Hospital, Karachi, Pakistan (reference number 2022–7178-21,416). Written informed consent was obtained from all the study participants. All methods were performed in accordance with the Declaration of Helsinki.

## Results

### Patient characteristics

In this study, 79 HNC treated patients who were within their first year of post-cancer treatment were included. Patients’ characteristics are shown in (Table 1). 51.9% (n = 41) participants out of total were below 52 years, 76% (n = 60) were males, showing a male predilection in the study population. 74.68% (n = 59) were married, 75.48%. (n = 60) had normal BMI while 21.52% (n = 17) participants were underweight. 55.7% (n = 44) were unemployed and 51.9% (n = 41) had monthly household income of PKR 50,000 or less.

**Table 1:** Sociodemographic, treatment related, habits, oral hygiene related factors of HNC patients within 1 year, post cancer treatment.

Variables	Frequency n (%)
**Age**	
< 52 years	41 (51.9%)
≥ 52 years	38 (48.1%)
**Gender**	
Male	60 (75.95%)
Female	19 (24.05%)
**Marital status**	
Married	59 (74.68%)
Unmarried	20 (25.32%)
**BMI**	
Underweight	17 (21.52%)
Normal weight	60 (75.48%)
**Tumor stage at diagnosis**	
Stage 1	10 (12.66%)
Stage 2	22 (27.85%)
Stage 3	46 (58.32%)
Stage 4	1 (1.27%)
**Type of the treatment received**	
Without radiotherapy	16 (20.25%)
With radiotherapy	63 (79.75%)
**Total dose of radiation received**	
< 33 cGy	21 (33.33%)
≥ 33 cGy	42 (66.66%)
**Elapsed time since the treatment**	
Less than 6 months	26 (32.9%)
6 months or more	53 (67.1%)
*Oral hygiene practices*	
**Brushing or use of applicator for teeth cleaning**	
Yes	49 (62.03%)
Seldom	21 (42.8%)
Once daily	18 (36.8%)
More than once daily	10 (20.4%)
No	30 (37.97%)
**Use of any mouthwash for oral hygiene**	
Yes	46 (58.23%)
No	33 (41.77%)
**Briefing about the oral complications of cancer treatment prior to start of treatment**	
Yes	48 (60.7%)
No	31 (39.24%)
**Instructions related to maintenance of oral hygiene by dental hygienist**	
Yes	36 (45.47%)
No	43 (54.43%)
** Referred to dental clinics for your oral complications during RT?**	
Yes	8 (10.13%)
No	71 (89.87%)
**Visited to dentist after completion of cancer treatment**	
Yes	23 (29.1%)
No	56 (70.89%)
**Use of any supplemental fluoride**	
Yes	23 (29.11%)
No	56 (70.89%)

At the time of diagnosis, 58.23% (n = 46) of the participants had stage III cancer, the majority comprising 79.75% (n = 63) received cancer treatment with radiotherapy along with either surgery or chemotherapy, while 20.2% (n = 16) underwent only surgery. The elapsed time after treatment for majority of the participants 67.1% (n = 53) was more than 6 months. 55.7% (n = 44) patients had attended more than three follow up visits at ENT clinics at Aga Khan University Hospital (AKUH), post treatment completion (Table 1).

### Oral hygiene practices among HNC treated patients

Out of participants, 62.03% (n = 49) reported regular teeth brushing, among these individuals, 42.86% (n = 21) reported infrequent daily teeth cleaning. Moreover, 70.89% (n = 56) mentioned that they were not prescribed any fluoride toothpaste or gel during or after cancer treatment. Of the study participants, the majority 60.7% (n = 48) received information about oral complications related to cancer treatment. Consequently, they exhibited a better understanding of associated oral symptoms, such as oral pain, mouth soreness, limited mouth opening and difficulties in eating and speaking recognizing that these concerns typically resolve over time. However, 54.4% (n = 43) of the participants did not receive specific instructions pertaining to the maintenance of oral hygiene during and after their cancer treatment, from either the consulting physician or dental hygienist. Furthermore, only a minor proportion, 10.1% (n = 8) of the participants sought dental consultation to address their oral complications following completion of cancer treatment (Table 1).

Among79 participants, 70.9% (n = 56) exhibited fair oral hygiene index score while 26.58% (n = 21) had poor oral hygiene score. Additionally, all participants included in the study experienced oral mucositis, 83.5% (n = 66) had moderate to severe oral mucositis upon examination (Table 2)*.*

**Table 2:** Oral clinical parameters i.e. OH status and OM status and comparisons of OHRQoL symptom scores of HNC patients within 1 year, post cancer treatment.

Variable	Frequency (%)	QLQ H&N- 35, mean (SD)
Oral hygiene index score		
Fair	21 (26.58%)	10.1 (7.8)
Poor	56 (70.89%)	16.2 (12.6)
Oral mucositis		
Mild	13 (16.46%)	23.3 (17.7)
Moderate to severe	66 (83.54%)	25.3 (15.5)

### OHRQoL and its associated factors

The study findings revealed a mean score for the oral health related quality of life (OHRQoL) was 25.02 ± 15.86 (95% CI 21.46–28.57)*.* Among the 35 items assessed, symptoms score for mouth opening (53.16 ± 18.88), dry month (45.14 ± 24.48), sexuality (39.71 ± 32.51) and speech (37.26 ± 17.61) exhibited higher values compared to other symptoms scores, indicating a more pronounced negative impact of cancer treatment on these functions. These factors collectively contributing significantly to the overall high OHRQoL score (Table 3)*.*

**Table 3:** Mean distribution of 18 domains scores of EORTC QLQ H&N -35 35 scores of HNC patients within 1 year, post cancer treatment.

Symptom score (n = 79)	Mean (SD)
PA (pain)	22.15 (16.28)
SW (swallowing)	17.40 (12.09)
PT (teeth)	19.83 (21.69)
MO (opening mouth)	53.16 (18.88)
DM (dry mouth)	45.14 (24.48)
SS (sticky saliva)	32.91 (24.16)
SE (senses)	9.915 (9.44)
CO (coughing)	19.40 (17.38)
FI (feeling ill)	21.94 (21.93)
SO (social eating)	23.31 (11.43)
SP (speech)	37.26 (17.61)
SC (social contact)	13.58 (13.64)
SX (sexuality)	39.71 (32.51)
PK (pain killers)	12.65 (16.28)
MV (supplements)	21.09 (16.16)
FT (feeding tube)	24.47 (4.810)
WL (weight loss)	13.92 (16.54)
WG (weight gain)	19.40 (16.54)

*High mean scores suggest more negative impact on OHRQoL.

Analysis of the OHRQoL scores, revealed noteworthy differences across demographic and health parameters. Females exhibited significantly higher score for difficulty in mouth opening (61.4 ± 16.7, p value 0.03) compared to males. Similarly, underweight patients demonstrated more adversely impacted OHRQoL (33.3 ± 13.8, p value 0.01) and experienced increased dry mouth symptoms (56.9 ± 22.9, p value 0.02) Patients with poor oral hygiene status displayed higher scores for OHRQoL (43.2 ± 15.8, p value 0.04) and dry mouth (66.6 ± 18.9, p value 0.03) (Table 4).

**Table 4:** Comparisons of OHRQoL symptom scores with few predicting factors of HNC patients within 1 year, post cancer treatment.

Variable	Mean (SD)		
	QLQ H&N- 35	MO	DM
**Gender**			
Male	23.33 (16.18)	50.55 (18.90)	43.33 (24.77)
Female	30.34 (13.88)	61.40 (16.71)	50.87 (23.22)
**BMI**			
Underweight	33.33 (13.81)	60.78 (13.09)	56.86 (22.86)
Not underweight	22.74 (15.72)	51.07 (19.75)	41.94 (24.1)
**Treatment type**			
Without radiotherapy	26.67 (18.58)	50 (24.34)	19.08 (27.13)
With radiotherapy	34.59 (15.23)	56.96 (17.38)	33.38 (23.67)
**Elapsed time since completion of treatment**			
Less than 6 months	27.11 (16.98)	55.13 (18.71)	50 (25.38)
Months or more	23.98 (15.34)	52.20 (19.1)	42.76 (23.90)
**Brushing frequency**			
Do not brush	27.43 (16.03)	58.89 (18.94)	47.77 (25.79)
Seldom	24.9 (14.41)	52.38 (16.90)	44.44 (19.24)
Once daily	18.74 (16.10)	35.55 (16.16)	31.85 (26.13)
More than once daily	11.28 (11.99)	33.33 (15.71)	26.66 (21.08)
**OHI score**			
Fair	17.68 (15.81)	36.54 (18.98)	28.21 (19.14)
Poor	26.19 (12.59)	42.85 (15.43)	34.92 (16.58)
**OM grade**			
Mild	23.3 (17.72)	48.7 (17.3)	43.59 (28.5)
Moderate—severe	25.3 (15.5)	54.04 (19.2)	45.45 (23.8)

High mean scores suggest a negative impact on OHRQoL.

*DM-dry mouth, *OHI score-Oral Hygiene Index score, *OM grade*-Oral Mucositis grade, *MO-mouth opening.

Regarding marital status, married patients had lower scores for OHRQoL scores and demonstrated better mouth opening compared to patients who were not married (see Table 5). Notably, individuals brushing teeth more than once daily had a significantly positive impact on OHRQoL (11.3 ± 4.57, p value 0.01), mouth opening (33.3 ± 15.7, p value < 0.01) and dry mouth (26.6 ± 11.9, p value 0.02) (Table 4).

**Table 5:** Factors associated with the OHRQoL in HNC patients within one year, post cancer treatment (multivariable model).

Variables	Adjusted β	95% CI	P-values
BMI (Ref = not underweight)			
Underweight	10.37	3.31–17.44	< 0.01
OHI score (ref = good)			
Fair	− 17.43	− 23.69– − 11.18	< 0.01
Poor	30.57	39.83–21.30	< 0.01
Marital status (ref = unmarried)			
Married	− 8.88	− 15.87– − 1.88	0.01
Monthly income (ref = < 50,000/- per month)			
≥ 50,000/- per months	− 6.69	− 0.72– − 12.67	0.02
Gender (Ref = female)			
Male	− 8.82	− 16.55– − 1.08	0.02
OM grade & fluoride toothpaste use			
OM grade (ref = mild), moderate + severe	9.42	− 1.79–20.63	0.09
Fluoride toothpaste use (ref = no), yes	− 14.9	− 1.66– − 28.15	0.02
OM grade # fluoride T/P	− 12.84	− 27.44–1.74	0.08

*CI* confidence interval, *BMI* = body mass index, *OM grade* oral mucositis grade.

# = Interaction.

*Adjusted β coefficient.

Table 5 shows that BMI, poor oral hygiene status, monthly income, oral mucositis grade were significantly associated with the decline in OHRQoL within one year, post cancer treatment. A significant interaction was observed in the effect model, showing a, lower mean score indicating better OHRQoL in patients with moderate to severe oral mucositis using fluoride toothpaste compared to patients with mild oral mucositis who were not using fluoride tooth paste (Table 5).

## Discussion

OHRQoL serves as an indicator of individual comfort with respect to their oral health while performing daily life activities such as eating, sleeping, socializing, etc. HNC patients often experience severe irreversible changes in the maxillofacial region post treatment, impacting crucial oral functions. Despite these changes, the influence on oral health quality of life (OHRQoL) remains insufficiently documented, especially in lower middle-income countries like Pakistan. The present study explored the impact of cancer treatment on oral health-related quality of life using EORTC QLQ-35 and the factors associated with it such as sociodemographic and clinical factors, in HNC treated patients who are within first year after completing their treatment. Our primary finding revealed a high mean score for OHRQoL (25.02 ± 15.86) among these patients. Marital status and gender showed associations with better OHRQoL, while low BMI and low monthly income were associated with poor OHRQoL.

Among previous investigations which have evaluated the OHRQoL in HNC patients^6,30^, this is the first study to explore the association between clinical characteristics such as oral hygiene practices, oral mucositis status and oral hygiene status and their impact on OHRQoL within 1-year of post cancer treatment using multivariable analysis. Consistent with prior research^31,32^, our study observed a negative impact on OHRQoL in our population Notably, 79% of the patients underwent surgery combined with chemo-radiotherapy (CTRT), significantly affecting their oral health and impairing oral functions like eating, speaking and maintaining oral hygiene practices, even a year after treatment completion.

Unlike previous studies from India, Sweden and Japan^6,30,33^ which reported symptoms like fatigue, weight loss, senses, sticky saliva and increased pain killer use as highly rated, our findings highlighted difficulty in mouth opening and dry mouth as most challenging symptoms significantly experienced by the HNC treated patients. These symptoms significantly contributed to lower OHRQoL comparable to a study in China^34^ Reduced salivary production by the salivary glands is a primary adverse effect of radiotherapy, leading to difficulty in chewing and swallowing along with pain and lesions in the oral cavity^35^ Another study reported a reduction in mean mouth opening from 45.58 mm to 42.55 mm at 6 months compared to baseline i.e. immediately after completion of cancer treatment^19^.

Varying degree of oral mucositis was found among all the patients included in the study, high score for OHRQoL 25.3 ± 15.6 (Table 2) was observed in patients with moderate to severe mucositis. These findings were consistent with a study conducted to evaluate the OHRQoL using the OHIP tool, which reported a significant difference in the OHRQoL score in patients with varying degrees of oral mucositis compared to patients without it^26^ Nevertheless, it is noteworthy that 52% of the patients experiencing moderate to severe OM symptoms had concluded their cancer treatment over six months ago. This finding is unexpected, given the studies have reported that symptoms of OM typically abate or become mild within a three-month period following cancer treatment^19,36^. A significant association between the oral mucositis severity and use of fluoride was also observed, suggesting improvement in the OHRQoL due to decrease in the severity of mucositis by the use of fluoride toothpaste, this is also supported by^37^ which recommended that the use of high fluoride tooth paste, fluoride gel or fluoride mouthwash prescribed to patients during and after their cancer treatment could reduce the severity of mucosal inflammation. This recommendation highlights that fluoride toothpastes are cost effective way to improve oral health post radiation treatment.

The current analyses showed a greater proportion of males (75.9%) compared to females (24.1%) similar to other studies^35,38^, which can be attributed to higher consumption of tobacco among males. The age group more susceptible to developing HNC is below 52 years (51.9%) correlating with other study by Chauhan et al.^39^ which assessed the prevalence and pattern of tobacco consumption before the development of HNC. However, patients aged above 52 years experienced a greater burden of oral complications following cancer treatment, which was evident through their significantly higher mean score for OHRQoL 26.4 ± 14.8 similar to the findings reported by Barma et al.^40^ and Lee et al.^41^ Participants who were underweight i.e. < 18.5 kg/m^2^ were more adversely affected with a OHRQoL score of 33.33 ± 13.81 which can be possibly due to the disturbance in daily eating and sleeping patterns because of oral complications, similar to findings reported by Huang et al.^42^ indicating significantly lowered OHRQoL in HNC patients who were underweight. Married participants had lower score of OHRQoL 22.9 ± 15.9 vs unmarried (30.34 ± 13.8) suggesting that patients with care takers may have a higher motivation to recover compared to those without partners or care takers^43^ in addition, loss of the partner or absence of the partner had a higher negative impact on OHRQoL which agrees with other study result^44^. Our study also suggested a significant association between low monthly income and decline in OHRQoL among HNC treated patients in Pakistan, which aligns with^45^ which has demonstrated that socioeconomic factors play a vital role in influencing the oral health of the patients. Also, low socioeconomic status prevents patients from seeking regular dental checkups and attaining specialized products to treat the oral complications post cancer treatment. This further strengthens the urgent need for public health initiatives aimed at improving oral care access and affordability for vulnerable populations in Pakistan.

Studies conducted in Korea^41^ and Taiwan^46^ have illuminated the significance of implementing comprehensive oral care programs in the context of head and neck cancer patients, with a focus on enhancing their overall quality of life (QoL). The impact of cancer treatment on oral health has been emphasized in multiple studies stating the challenges caused by the impairment of the oral functions plays pivotal role in the deterioration of the OHRQoL of patients treated with HNC. Our own results findings as presented in Table 4, align with these established patterns, underscoring the substantial role of mouth opening limitation in contributing to the decline in OHRQoL. In the context of Pakistan, it is essential to highlight the scarcity of comprehensive oral care programs, a situation where cancer patients receive minimal or no guidance on maintaining their oral hygiene. According to a study, regular visits to the dentist and professional oral hygiene care during the first 8 weeks of the radiotherapy were effective for maintaining oral health of HNC patients^47^. Our research revealed that 54.4% of the patients did not receive any guidance or instructions from either the dental hygienist to mitigate the oral complications arising after the cancer treatment, merely 10% of the patients were directed to dental clinics in response to the severity of their complication and rehabilitation needs, 29% patients took the initiative to visit dental clinics independently. This deficiency has resulted in patients enduring substantial distress even after the completion of the treatment, suggesting the imperative significance of oral care programs by proactively addressing the distressing symptoms like dry mouths and swallowing difficulties through tailored interventions such as oral hygiene practices, utilization of substitute saliva and rehabilitation exercises, enhancing OHRQoL.

Some of the study limitations that warrants discussion were, firstly, this study adopted a cross-sectional design, thus providing a glimpse in the OHRQoL of HNC treated patients at a particular moment making it challenging to analyze long-term trends over time. Secondly, it is noteworthy that the research was conducted exclusively within a single center in Pakistan, as a result, caution should be exercised when extending the findings to a broader context or diverse population as the generalizability of the result may be restricted. We suggest that future research involving multiple hospitals, recruiting participants from various regions within Pakistan, would capture a diverse perspective of patient experience related to their oral health, post cancer treatment. Additionally, comparative studies across different countries can highlight the influence of oral care programs and other related factors on OHRQoL among individuals treated for HNC. Another limitation is that it is important to acknowledge that the data collection relied on self-reported information introducing the possibility of result overestimation. However, the potential bias was mitigated through the rigorous training of the data collector ensuring that the patients’ interviews were conducted in a manner conducive to maximize the reliability of the responses.

### Implication for practice

This research emphasizes the critical need to incorporate comprehensive oral care protocols into the treatment of the head and neck cancer patients in Pakistan. Education including regular brushing with fluoride toothpaste and judicious use of gentle mouthwash and awareness initiatives are essential to inform patients about the potential oral side effects of the treatment they are taking, emphasizing good oral hygiene practices. Consistent monitoring by the dental professionals is crucial to manage acute oral complications, while interdisciplinary collaboration among healthcare teams is vital to address the multifaceted challenges faced by the HNC patients post treatment. Notably, the study highlights the potential benefits of fluoride toothpaste in mitigating mucositis severity and improving oral health. There is a need to develop and appraise oral care initiatives tailored for individuals affected by oral cancers by conducting longitudinal research to track oral health evolution aiming to enhance the quality of life and overall well-being of HNC patients in Pakistan.

## Conclusion

The study findings reveal a substantial negative impact on the oral health related quality of life among HNC treated patients, particularly those who underwent radiotherapy. This negative impact is more pronounced in patients experiencing difficulty in mouth opening and dry mouth caused by hypo salivation. These findings highlight the need to reevaluate the current treatment approaches such as refining treatment protocols through advanced radiation techniques, tailored treatments to each patient’s unique needs and characteristics in terms of health and preferences, better education about treatment process, potential side-effects, involving multidisciplinary teams including oncologist, surgeons, radiation therapist, dentist, speech therapist and mental health professionals to provide a more holistic approach to patient care and integration of oral care programs as a part of HNC treatment to mitigate the adverse effects of treatment on oral heath, to improve OHRQoL and overall QoL in HNC treated patients.

## Data Availability

The datasets generated and analyzed during the current study are not publicly available due to individual privacy could be compromised but are available from the corresponding author on reasonable request.

## Abbreviations

HNC: Head and neck cancer
OHRQoL: Oral health related quality of life
QoL: Quality of life
EORTC QLQ H&N: European organization of research and treatment of cancer, quality of life questionnaire
OHI-s: Oral hygiene index-simplified
OM: Oral mucositis
CT: Chemotherapy
RT: Radiotherapy
JCIA: Joint Commission International Accreditation
